# Association between male infertility and either the +331G/A or the progins polymorphism of the progesterone receptor gene in a Chinese population

**Published:** 2015-01

**Authors:** Dandan Li, Junjie Cheng, Wanghong Li, Wei Ma, Xu Zhou, Lianwen Zheng, Chunjin Li

**Affiliations:** 1 *The Second Hospital of Jilin University, Reproductive Medical Center, Changchun, Jilin Province, 130062, China.*; 2 *College of Animal Sciences, Jilin University, 5333 Xi’an Avenue, Changchun, Jilin Province, 130062, China.*; 3 *College of Animal Science and Veterinary Medicine, Liaoning Medical University, Jinzhou, Liaoning, China.*

**Keywords:** *Progesterone receptor*, *+331G/A polymorphism*, *Progins polymorphism*, *Male infertility*

## Abstract

**Background:**

Progesterone has been suggested to contribute to the regulation of spermatogenesis and to facilitate the production of viable sperm. Investigations have showed that polymorphism of progesterone receptor (PGR) is associated with some diseases.

**Objective:**

To analyze the potential relationship between male infertility and the +331G/A and progins polymorphisms of PGR gene.

**Materials and Methods:**

The cross-sectional study was carried out at the Department of Male Reproduction, Reproductive Medical Center, the Second Hospital of Jilin University. The restriction fragment length polymorphism (RFLP) technique was used to detect gene point mutations. Of the 145 semen samples analyzed, 35 were asthenozoospermic, 50 were oligoasthenozoospermic, 21 were azoospermic, 11 were teratozoospermic and 28 were from fertile male subjects.

**Results:**

Statistical analyses revealed that the genotypes of the +331G/A polymorphisms were in Hardy-Weinberg equilibrium in both the fertile (^2^=0, p=0.534) and oligospermic groups (^2^=0.021, p=0.537). Similarly, the genotypes of the progins polymorphisms were also in Hardy–Weinberg equilibrium in both the fertile (^2^=0, p=1) and oligospermic groups (^2^=0.005, p=1).

**Conclusion:**

Our results indicated that polymorphisms of the +331G/A and progins of the PGR gene are unrelated to male infertility, at least in a Chinese population.

## Introduction

Around 15% of couples worldwide suffer from fertility problems, with male infertility accounting for around 50% of these cases (1). There is therefore considerable academic interest in the genetic epidemiology of male infertility. Specific genetic abnormalities that have been linked to male infertility include the USP9Y, Kif18a, and RBMY deletions, as well as spot mutations in the 5-methylenetetrahydrofolate reductase (MTHFR) (C677T) and DAZL genes (2-8).

It has been suggested that the progesterone mRNA is expressed in the male sexual glands. In addition, membrane-bound progesterone receptor (PGR) are known to be present in testicular cells (9). The binding of progesterone to its receptor has been suggested to contribute to the regulation of spermatogenesis and to facilitate the production of viable sperm. This implies that some cases of male infertility may occur due to deficiencies in PR expression, which would cause insensitivity to progesterone. Significant correlations between polymorphisms in the PGR gene and diverse human diseases have previously been identified. 

One of the most notable polymorphisms in this group is a 306 bp indel known as progins, which is located within intron 7 of the PGR gene. It has been reported that in women, the progins polymorphism is associated with endometriosis, an increased likelihood of preterm labor, and the formation of uterine fibroids (10, 11). In addition, the +331G/A polymorphism of PR has been linked to cancers of the female reproductive system, although no obvious correlation with the occurrence of breast cancer was identified (12, 13). PR polymorphic variant, G to A exchange at position +331 of the promoter region, which alter either function or expression of PR. This single nucleotide polymorphism (SNP) results in the introduction of a TATA-box, which exclusively enhances transcription of PR-B thereby increasing the ratio PR-B to PR-A (11).

To date, there have been no reports on the potential links between male infertility and polymorphisms of PGR gene. The aim of the present study was to investigate whether there is any relationship between male infertility and two known PGR gene polymorphisms: progins and +331G/A.

## Materials and methods

In this cross-sectional study, all experiments were carried out at the Department of Male Reproduction, Reproductive Medical Center, The Second Hospital of Jilin University in the year of 2012. All reagents used in the study were purchased from commercial companies. Written informed consent was obtained from all participants in the study, and the study was approved by the National Medical Ethics Committee.

Blood samples were collected from male subjects who were recruited at random. All samples were mixed with anticoagulants and stored in test tubes at -20^o^C until required for analysis. Semen samples were obtained from each participant by masturbation after 2-5 days of sexual abstinence and allowed to liquefy for 30 min at 38.5^o^C before further processing. Semen analysis was performed according to the routine semen inspection standard published by the World Health Organization (1987). In total, 145 individuals provided blood and semen samples. 

Of the 145 semen samples analyzed, 35 were asthenozoospermic (<25% rapid motility or <50% progression in a semen sample, and fresh sperm concentration >20x10^6^ ml), 50 were oligoasthenozoospermic (motility <25%, and fresh sperm concentration <20x10^6^ ml), 21 were azoospermic (>25% rapid motility or <50% progression in a semen sample, and fresh sperm concentration >20x10^6^ ml), 11 were teratozoospermic and 28 were from fertile male subjects (>25% rapid motility or >50% progression in a semen sample, and fresh sperm concentration >20x10^6^ ml). Purification of genomic DNA from the human blood samples was performed using a kit obtained from AXYGEN (Hangzhou, China) according to the manufacturer’s recommendations. Two pairs of primers were designed to detect the PGR +331G/A (Forward: CACTACTGGGATCTGAGATC; Reverse: CACAAGTCCGGCACTTGAGT) and progins (Forward: GCCTCTAAAATGAAAGG CAGAAAG; Reverse: AAAAGTATTTTCTTG CTAAATGTCT) polymorphisms. 

Different amplification by the polymerase chain reaction (PCR) was conducted in reaction volumes of 25 μl containing 1μl of sample DNA, 2.5 μl of 10×Buffer, 0.5μl of each primer (PGR +331G/A and progins were amplified in separated tube), and 0.20 units of rTaq polymerase (TaKaRa Biotechnology). The amplification products were purified using a kit supplied by ShengGong (Shanghai, China) and the purified products were sent to Shanghai ShengGong for sequencing. The PCR products obtained using the primers targeting the +331G/A polymorphism were digested with 10μl of BspLI at 37^o^C for 3 hr. 

The resulting DNA fragments were then separated by electrophoresis on a 0.2% agarose gel and visualized after ethidium bromide staining. The amplification products obtained using the primers for the progins polymorphism were similarly separated and analyzed by electrophoresis.

**Statistical analysis**

The presence of Hardy-Weinberg equilibrium for the genotypic distribution was tested by using the  ^2^ test for goodness-of-fit. Genotype and allele frequencies between patients and control subjects were compared by Pearson’s Chi-squared test. Odds ratio (OR) and 95% confidence interval (95% CI) were used to express the relative risk. Calculations were performed using the Statistical Package for the Social Sciences, version 17.0, SPSS Inc, Chicago, Illinois, USA (SPSS). 

## Results

**Figure 1:**
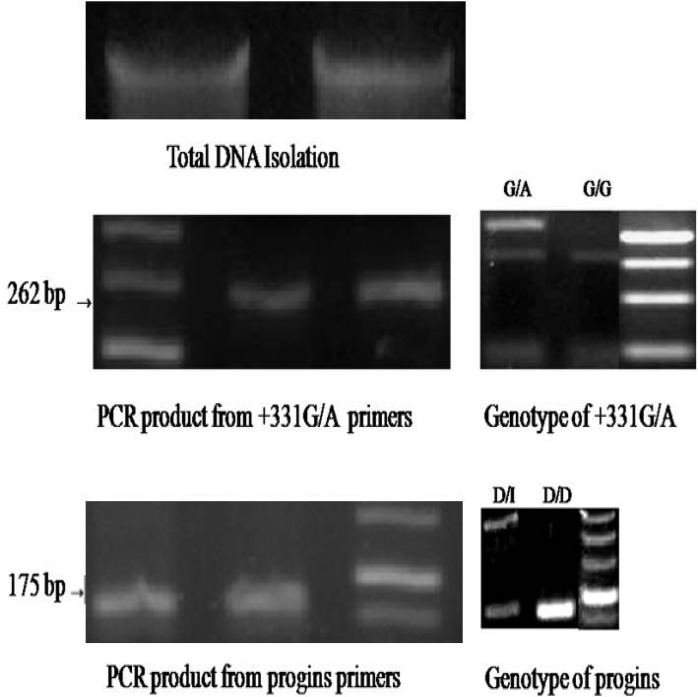
Gel electrophoresis of DNA isolation, PCR products and genotyping. Total DNA were isolated from blood samples of male subjects (A). A fragment of 262 bp was amplified using PGR +331G/A primers, and +331G/A polymorphism were analyzed with BspLI digestion (G/A, G/G) (B). A fragment of 175 bp was amplified using progins primers, and a 306 bp insert was detected (D/I, D/D) (C).

Some of the sampled individuals had the G/A single nucleotide polymorphism at the +331G/A site in the PGR gene, only two of the infertile subjects (both of whom suffered from oligospermia) had the A/G genotype; all of the other subjects were G/G homozygous. In addition, while the 306 bp insertion/deletion progins polymorphism was detected in the PGR gene of the test subjects, only two individuals with oligospermia had the I/D hybrid genotype There were no I/I homozygous individuals among the test subjects (Table I, Figure 1). 

Statistical analyses revealed that the genotypes of the +331G/A polymorphisms were in Hardy–Weinberg equilibrium in both the fertile ( ^2^=0, p=0.534) and oligospermic groups ( ^2^=0.021, p=0.537). Similarly, the genotypes of the progins polymorphisms were also in Hardy–Weinberg equilibrium in both the fertile ( ^2^=0, p=1) and oligospermic groups ( ^2^=0.005, p=1). 

The genotype distributions of the +331G/A and progins polymorphisms in the oligoasthenozoospermic and fertile groups are shown in tables II, III. There were no significant differences in the genotype distributions for the oligoasthenozoospermic and fertile groups.

**Table I:** Genotyping data of +331G/A and progins for the 145 test subjects

**Sample (n)**	**+331G/A**				**Progins**	
	G/G	A/A	G/A	D/D	I/I	D/I
Fertile (28)	28	0	0	28	0	0
Oligoasthenozoospermic (50)	48	0	2	49	0	1
Asthenozoospermic (35)	35	0	0	35	0	0
Azoospermic (21)	21	0	0	21	0	0
Teratozoospermic (11)	11	0	0	11	0	0

**Table II:** Distribution of genotypes and alleles frequencies of +331G/A for fertile and infertile subjects

**Sample (n)**		**Genotype**				**Allele**	
		**G/G**	**G/A**	**A/A**		**G**	**A**
							
Fertile (28)		28 (100.00)	0 (0.00)	0 (0)		56 (100.00)	0 (0.00)
Oligoasthenozoospermia (50)		48 (96.00)	2 (4.00)	0 (0)		98 (98.00)	2 (2.00)
p-value			0.534			0.537	

Genotype and allele frequencies between patients and control subjects were compared by Pearson’s chi-squared test. p<0.05 was considered statistically significant.

**Table III:** Distribution of genotypes and alleles frequencies of progins for fertile and infertile subjects

**Sample (n)**		**Genotype**				**Allele**	
		D/D	D/I	I/I		D	I
Fertile (28)		28 (100.00)	0 (0.00)	0 (0)		56 (100.00)	0 (0.00)
Oligoasthenozoospermia (50)		49 (98.00)	1 (2.00)	0 (0)		99 (99.00)	1 (1.00)
p-value			1.00			1.00	

Genotype and allele frequencies between patients and control subjects were compared by Pearson’s chi-squared test. p<0.05 was considered statistically significant.

## Discussion

The analysis of genetic polymorphisms is a powerful tool for studying the causes of male infertility. Several polymorphisms have been shown to correlate with specific types of male infertility, including polymorphisms in the number of CAG repeats in exon 1 of the androgen receptor (AR), FSHR, estrogen receptor (ER) α, and gr/gr deletions in the *AZFc* gene (14-16). The findings in these areas to date have been reviewed and subjected to meta-analysis (17, 18). 

Polymorphisms in the human PGR gene have been linked to many diseases in women, including conditions that affect fertility. It has also been shown that polymorphisms in the PGR gene are linked to semen quality in some animals. Progesterone receptor has been detected in human sperm. Both immunocytochemical method and cell-impermeable fluorescei-tagged progesterone coupled to BSA complex (P-FITC-BSA) showed that expression of PGR in men with oligozoospermia, asthenozoospermia, and oligoasthenozoospermia was significantly lower than that of men with normal spermatozoa, indicating that PGR might be a potential marker for sperm function (19). 

It therefore seemed plausible that polymorphisms in the human PGR gene might also affect male fertility; in particular, it has been suggested that some cases of male infertility may be due to deficiencies in the expression of the PGR and the resulting insensitivity to progesterone. We therefore sought to determine whether known mutations in the PGR gene might reduce PGR expression and affect fertility in men. 

Our results indicate that there were no significant differences in genotype or allele frequency with respect to the +331G/A and progins polymorphisms between the infertile and control groups examined in this work. The failure to identify significant differences may have been due to the small size of the subject group or may indicate that this region is highly conserved. Because only two polymorphic loci within the PGR gene were considered, we cannot conclude with certainty that there is no relationship between polymorphisms in PGR and male infertility. 

Other study also showed that four SNPs in PGR gene (rs3740753, rs1042838, rs104283, and progins) were not associated with male infertility. Further research using a larger subject group and a broader range of candidate loci will therefore be required to fully explore the potential effects of PGR polymorphisms on male (in) fertility. Until now, there is no direct evidence about PGR in spermatogenesis. Both mice models with conditional knock out (in) PGR in testis and cell culture models are very necessary to explore the role of PGR in testicular development and spermatogenesis.

## Note

***This article extracted from M.Sc. thesis. (Junjie Cheng)***
